# Applications of Synthetic Pentatricopeptide Repeat Proteins

**DOI:** 10.1093/pcp/pcad150

**Published:** 2023-11-30

**Authors:** Farley Kwok van der Giezen, Suvi Honkanen, Catherine Colas des Francs-Small, Charles Bond, Ian Small

**Affiliations:** Australian Research Council Centre of Excellence in Plant Energy Biology, School of Molecular Sciences, The University of Western Australia, 35 Stirling Highway, Perth, WA 6009, Australia; Australian Research Council Centre of Excellence in Plant Energy Biology, School of Molecular Sciences, The University of Western Australia, 35 Stirling Highway, Perth, WA 6009, Australia; Australian Research Council Centre of Excellence in Plant Energy Biology, School of Molecular Sciences, The University of Western Australia, 35 Stirling Highway, Perth, WA 6009, Australia; School of Molecular Sciences, The University of Western Australia, 35 Stirling Highway, Perth, WA 6009, Australia; Australian Research Council Centre of Excellence in Plant Energy Biology, School of Molecular Sciences, The University of Western Australia, 35 Stirling Highway, Perth, WA 6009, Australia

**Keywords:** Chloroplasts, Mitochondria, Pentatricopeptide repeat (PPR) proteins, RNA-binding proteins, RNA editing

## Abstract

RNA-binding proteins play integral roles in the regulation of essential processes in cells and as such are attractive targets for engineering to manipulate gene expression at the RNA level. Expression of transcripts in chloroplasts and mitochondria is heavily regulated by pentatricopeptide repeat (PPR) proteins. The diverse roles of PPR proteins and their naturally modular architecture make them ideal candidates for engineering. Synthetic PPR proteins are showing great potential to become valuable tools for controlling the expression of plastid and mitochondrial transcripts. In this review, by ‘synthetic’, we mean both rationally modified natural PPR proteins and completely novel proteins designed using the principles learned from their natural counterparts. We focus on the many different applications of synthetic PPR proteins, covering both their use in basic research to learn more about protein–RNA interactions and their use to achieve specific outcomes in RNA processing and the control of gene expression. We describe the challenges associated with the design, construction and deployment of synthetic PPR proteins and provide perspectives on how they might be assembled and used in future biotechnology applications.

## Introduction

RNA maturation and regulation in eukaryotes is facilitated by a diverse set of RNA-binding proteins (RBPs). Over 2,700 proteins have been implicated in RNA binding in plants (Marondedze 2020), with diverse roles in RNA regulation and expression dependent on a variety of factors including different growth conditions and environmental stresses (Burjoski and Reddy 2021). RBPs typically contain one or more RNA-binding domains. Some common RNA-binding domains in the *Arabidopsis thaliana* mRNA interactome are RNA recognition motifs, helicase core domains, Q-motifs of DEAD box helicases and zinc finger and pumilio domains (Marondedze et al. 2016). Much of the work in engineering RBPs has focused on the use of the bacterial RBP Cas13 fused to functional domains such as adenosine deaminase acting on RNA to edit RNAs or degrade viral RNA via RNA interference (Kavuri et al. 2022). Cas13 is effective for targeting nuclear and cytoplasmic transcripts but is limited in its ability to affect organelle transcripts due to the difficulty of importing guide RNAs across organelle membranes (Yoo et al. 2020). Pentatricopeptide repeat (PPR) proteins constitute the largest RBP family in plant organelles (Barkan and Small 2014). PPR motifs recognize RNA bases according to the identities of two key amino acids (aas) in the PPR motif. A binding code developed from studying these interactions (Barkan et al. 2012, Yagi et al. 2013, Kobayashi et al. 2019, Yan et al. 2019) has allowed for the design of synthetic PPR proteins with predictable and modifiable RNA binding capabilities (Coquille et al. 2014, Shen et al. 2015, Yan et al. 2017, Bernath-Levin et al. 2021, Royan et al. 2021, Ichinose et al. 2022). A major benefit of synthetic PPR proteins is that RNA binding specificity is encoded in the protein and is thus not reliant on guide RNAs. Our ability to control plastid and mitochondrial transcripts using engineered PPR proteins is rapidly growing, and thus, synthetic PPR proteins have great potential to become valuable biotechnology tools for engineering regulation of plastid and mitochondrial transcripts. Methods for constructing synthetic PPR proteins have been reviewed recently (McDowell et al. 2022), so here, we focus instead on demonstrated and potential applications of synthetic PPR proteins.

## PPR Proteins

PPR genes were first identified in the genome of *A. thaliana* as encoding proteins containing tandem arrays of 35-aa motifs related to the tetratricopeptide repeat (TPR) (Small and Peeters 2000). Subsequently, PPR proteins have been shown to play key roles in organelle transcript processing including transcript stabilization (Pfalz et al. 2009, Ruwe and Schmitz-Linneweber 2012, Zhelyazkova et al. 2012, Rojas et al. 2018), RNA cleavage (Gobert et al. 2010, Binder et al. 2013, Zhou et al. 2017, Melonek et al. 2021), RNA splicing (Schmitz-Linneweber et al. 2006, Chateigner-Boutin et al. 2011, Aryamanesh et al. 2017, Lee et al. 2019), RNA editing (Kotera et al. 2005; Small et al. 2020, Knoop and Marquardt 2023) and translational activation (Prikryl et al. 2011, Zoschke et al. 2016). PPR proteins form a superhelical structure with an internal RNA-binding groove that associates with RNA in a parallel orientation with each PPR motif bound to a single RNA nucleotide (Fujii et al. 2011). Each motif recognizes a specific RNA base primarily according to the identities of aas at the fifth and last position within the motif (Barkan et al. 2012). Broadly, PPR proteins are divided into two groups according to their motif structures and organization; these are the P-class and PLS-class sub-groups (Cheng et al. 2016).

**Fig. 1:**
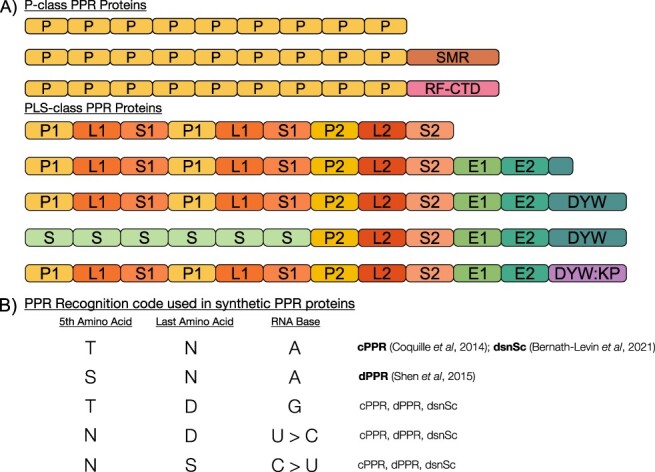
(A) PPR repeat proteins are divided into two classes according to the architecture of their PPR motif array which binds to RNA. P-class PPR proteins are made of tandem repeats of a 35-aa PPR motif, and PLS-class proteins are mostly RNA editing factors made of triplets of P-, L- and S-type PPR motifs, which vary in length, followed by PPR-like E1 and E2 motifs. Various C-terminal domains may be appended to PPR proteins, often conferring specific functionality. The examples shown are the SMR domain, restorer-of-fertility C-terminal domain, cytidine deaminase domain (DYW) and uridine aminase domain (DYW:KP). (B) PPR proteins recognize RNA bases largely through interactions between aas at the fifth and last position in PPR motifs. In nature, many combinations of aas are observed. Synthetic PPR proteins and motifs use a PPR code based on the strongest and most specific interactions between fifth and last aas and their associated RNA base.

P-class PPR proteins are characterized by an array of tandem repeats of the canonical 35-aa P-type PPR motif (Lurin et al. 2004, Cheng et al. 2016) (**Fig. 1A**). They are involved in many RNA processing steps in chloroplasts and mitochondria, such as protecting transcripts against 5′ or 3′ exonucleolytic digestion (Beick et al. 2008, Pfalz et al. 2009, Haïli et al. 2013), increasing translation efficiency by guiding RNA unfolding (Prikryl et al. 2011, Zoschke et al. 2013, 2016) and group II intron splicing (Falcon de Longevialle et al. 2007, Lee et al. 2017, 2019). Some P-class PPR proteins contain C-terminal domains that confer specific functionality, e.g. PPR-small MutS-related (SMR) proteins contain a SMR C-terminal domain that is putatively involved in RNA cleavage (Liu et al. 2013, Zhou et al. 2017). Restorer-of-fertility and restorer-of-fertility-like (RFL) PPR proteins are P-class PPRs that induce cleavage of specific mitochondrial transcripts (Binder et al. 2013, Huynh et al. 2023). Deletion of the C-terminal domain of RFL proteins abolishes the cleavage (Huynh et al. 2023), suggesting that this domain is involved in endonuclease recruitment.

Almost all PLS-class PPR proteins are RNA editing factors. PLS-class PPRs differ from P-class PPRs in their PPR motif arrays arranged in triplets of P1 (35 aa), L1 (35–36 aa) and S1 (31 aa) motifs (**Fig. 1A**). PLS-class PPRs generally have a motif organization of (P1–L1–S1)*_n_*–P2–L2–S2 (Cheng et al. 2016). The P2–L2–S2 motifs have diverged from the P1–L1–S1 motifs (Rivals et al. 2006) and differ slightly in their preferred aa residue and nucleotide interactions (Cheng et al. 2016). The C-terminal domain of many PLS-class PPR proteins is defined by two PPR-like motifs E1 (34 aa) and E2 (34 aa), which precede a 135–136 aa cytidine deaminase–like domain (known as the DYW domain) responsible for catalyzing C-to-U RNA editing (Salone et al. 2007, Oldenkott et al. 2019, Takenaka et al. 2021). The DYW:KP domain is a variant of the DYW domain that is present in hornworts, lycophytes and ferns (Gerke et al. 2020, Gutmann et al. 2020). The DYW:KP domain has been demonstrated to catalyze U-to-C editing, which is unique to hornwort, lycophyte and fern plastid and mitochondrial transcripts (Ichinose et al. 2022). In many PLS-class PPRs, the DYW domain is truncated or absent, but can be supplied in *trans* via interaction with another PPR protein (**Fig. 1A**). Examples of PPR editing factors that rely on this type of protein–protein interaction to achieve editing are *A. thaliana* CRR4 and CLB19 (Kotera et al. 2005, Chateigner-Boutin et al. 2008, Boussardon et al. 2012, Andrés-Colás et al. 2017, Guillaumot et al. 2017). In angiosperms, PLS-class PPR proteins bind their RNA targets together with co-factors such as multiple organellar RNA editing factor (MORF) [or RNA editing interaction proteins (RIP)], organelle RNA recognition motif proteins and organelle zinc finger proteins (Sun et al. 2016). In contrast, the PLS-class PPR editing factors from moss *Physcomitrium patens* can bind and edit their target RNAs in vitro, in *Escherichia coli* and in human cell cultures without these co-factor proteins, of which at least MORF proteins are not found in seed-free plants (Schallenberg-Rüdinger and Knoop 2016, Oldenkott et al. 2019, Gutmann et al. 2020, Hayes and Santibanez 2020, Lesch et al. 2022). RNA editing PPR proteins are particularly attractive targets for engineering due to their potential for altering protein-coding sequences or translational control elements.

## The PPR Code

A convenient and extremely useful description of how PPR proteins recognize their target RNAs (‘the PPR code’) was developed by aligning PPR proteins with known RNA-binding sites (Barkan et al. 2012, Yagi et al. 2013). A prerequisite for these efforts was the finding that PPR proteins align in parallel orientation to the RNA, unlike PUF proteins that align in antiparallel orientation (Filipovska et al. 2011), and that each PPR motif probably contacts a single base in the RNA (Fujii et al. 2011). Furthermore, contrasting evolutionary patterns between PPR proteins under purifying or diversifying selection and structural modeling indicated which aas in the PPR motifs were most likely interacting with the RNA and likely to determine binding specificity (Fujii et al. 2011). The first PPR code was described in a study of the maize chloroplast P-class PPR protein PPR10, which binds as a monomer to 5ʹ untranslated regions of the plastid *psaJ* transcript (Barkan et al. 2012). Barkan et al. observed a pattern of asparagine (N) which is now generally referred to as the fifth position of the PPR motif generally aligned with cytidine and uridine, serine (S) or threonine (T) aligned with adenine and guanidine, and aspartic acid (D) at the last aa position aligned with uridine (**Fig. 1B**). A nearly identical PPR code for P- and S-type PPR motifs was in parallel identified by Yagi et al. (2013) by aligning 32 RNA editing PPR proteins with known editing sites to 5′ *cis* regions of their editing sites and then comparing the correlation of aas in the fifth and last position with their associated nucleotides (Yagi et al. 2013). Crystal structures of PPR10 bound or unbound to its RNA targets provided the structural confirmation of the molecular recognition of the RNA bases A, G and U by PPR motifs (Yin et al. 2013). The ‘PPR code’ has since been refined by the addition of more data and more sophisticated data analysis (Takenaka et al. 2013, Harrison et al. 2016, Kobayashi et al. 2019). This code has been invaluable for predicting the targets of natural PPR proteins, but in the context of this review, it was a crucial prerequisite to the development of synthetic PPR proteins as it permitted the design of proteins aimed at chosen target sites.

## Synthetic PPR Proteins

### The use of synthetic PPR proteins in structural studies

**Fig. 2:**
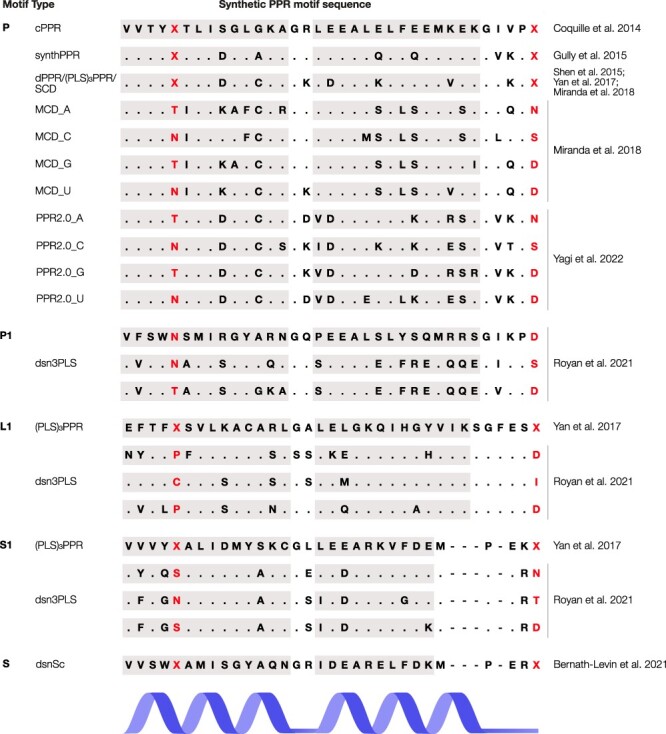
Synthetic PPR motif designs based on consensus sequences of aligned PPR motifs. Fifth and last aas strongly contribute to the RNA base preference of PPR motifs and are highlighted. The letter ‘X’ denotes instances where multiple fifth and last aa combinations were tested. Identical residues between the synthetic PPR motifs have been replaced with a period. Residues missing from the shorter S-type motifs relative to P- and L-type motifs have been replaced with a hyphen. The alignment of the motifs corresponds to the PPR motifs defined in Cheng et al. (2016). The regions predicted to fold into alpha helices in Cheng et al. (2016) are shaded in gray.

Synthetic PPR proteins have been instrumental in developing our understanding of the structure of PPR proteins and how they interact with their RNA targets. Initial attempts to solve structures of PPR proteins were hindered by the poor solubility of natural PPR proteins when expressed in *E. coli*. The first PPR structure to be solved was a truncation of *Zea mays* PPR10 with quadruple cysteine (C) to serine (S) mutations to avoid formation of disulfide bonds and increase protein solubility (Yin et al. 2013). The first synthetic PPR proteins were designed by several groups in parallel for use in structural and functional studies (Coquille et al. 2014, Gully et al. 2015b, Shen et al. 2016). All used a similar consensus design strategy that had previously been used for other proteins including TPR proteins, which are distantly related to PPR proteins but generally involved in protein–protein interactions (Main et al. 2003a, 2003b, Kajander et al. 2006, 2007). Coquille et al. designed a synthetic P-class PPR protein, called ‘consensus PPR’ (cPPR), which was derived from a multiple sequence alignment of 23,916 PPR sequences, whereas the ‘synthPPR’ in the study by Gully et al. was derived from a profile hidden Markov model generated from 2,357 PPR motifs found in *A. thaliana*. ‘dPPR’ synthetic PPR motifs in the study by Shen et al. were also based on multiple sequence alignments of *A. thaliana* P-class PPR motifs. In each case, the most representative aa at each position (1–35) was used to create a synthetic PPR motif, except for cysteine at position 12, which was substituted with glycine (G) (Coquille et al. 2014) or alanine (A) (Gully et al. 2015b) to avoid disulfide bond formation, although Shen et al. retained C12 in their ‘dPPR’ consensus motif. Gully et al. also substituted two out of the five negatively charged glutamic acids (E) at the solvent-exposed face of helix β to neutral glutamine (Q), as very few native PPR motifs were observed to have more than three negatively charged residues in this region. Overall, the first three synthetic P-type PPR motif scaffolds differed in 9 out of the 35 aa positions (**Fig. 2**).

Each synthetic PPR protein design also used different N- and C-terminal caps around the PPR motif tract to improve stability and solubility. The N- and C-terminal sequences in dPPR protein design in the study by Shen et al. were derived from the natural maize PPR protein PPR10 (N-terminal aas 37–208 and C-terminal aas 737–786). cPPR in the study by Coquille et al. contained eight PPR motif repeats flanked by an N-terminal cap sequence (Met-Gly-Asn-Ser) derived from naturally abundant aas at N-terminal positions in α-helices as described in Richardson and Richardson (1988) and a C-terminal solvating helix used previously in synthetic TPR proteins to prevent protein unfolding (Main et al. 2003b). Gully et al. used a different N-terminal cap sequence (Ala-Gly-Met-Asn) from Dasgupta and Bell (1993) and an additional helix A from their synthPPR design, with four substitutions (Y5N, I9K, L12A and A13S) to create an amphipathic C-terminal solvating helix. Similar to the synthetic TPR proteins created using the consensus design strategy, synthetic PPR proteins were observed to have superior solubility and thermal stability compared to natural PPR proteins (Main et al. 2003b, Kajander et al. 2007, Coquille et al. 2014, Shen et al. 2015).

Using their respective synthetic PPR motif designs, Coquille et al., Gully et al. and Shen et al. were able to purify and crystallize several synthetic PPR proteins, each with altered target specificity as a result of modifying the 5th and 35th aa identity according to the PPR code (Yagi et al. 2013). Coquille et al. purified and crystallized four different ‘cPPR’ proteins, each targeting a different RNA sequence of polyA, polyC, polyG and a nanos response element (NRE) RNA sequence; Gully et al. produced two different lengths of synthPPR with 3.5 and 5.5 motifs; Shen et al. purified and crystallized four different 10-motif ‘dPPR’ proteins targeting a sequence of 5′-UUUUNNUUUU-3′, with N denoting different nucleotide pairs (AA, GG, CC, UU). Neither Coquille et al. nor Gully et al. were able to solve the structure of their synthetic PPR proteins with molecular replacement using atomic coordinates from the native PPR protein structures available at the time (mtRNAP, PPR10, PRORP1 and THA8) (Ringel et al. 2011, Howard et al. 2012, Ke et al. 2013, Yin et al. 2013), and instead, both used anomalous data measured from selenomethionine derivatives of their proteins. Shen et al. were able to use the atomic coordinates of the cPPR targeting NRE in the study by Coquille et al. to solve the structure of their dPPR proteins. Coquille et al. and Gully et al. noted that the synthetic PPR proteins had more consistent intra- and inter-motif angles than natural PPR proteins.

The first PLS-class synthetic PPR protein was designed by Yan et al. (2017) and utilized the P-motif from the dPPR synthetic motif in the study by Shen et al., as well as consensus L- and S-type motifs derived from 263 *A. thaliana* L-type motifs and 1,117 S-type motifs (**Fig. 2**). Yan et al. included an N-terminal cap and a C-terminal solvating helix sequence from maize PPR10 to create a synthetic PLS-class PPR protein with three triplets of P–L–S motifs, called (PLS)_3_PPR. Purification and crystallization of (PLS)_3_PPR revealed a superhelical structure similar to previous synthetic PPR protein structures. Yan et al. measured the distances between the fifth aa and its associated RNA base in each of the motifs and found that the distance between the last aa of the L-motif and its associated RNA base was twice as large (6.03 Å) relative to P- and S-type motifs (3.02 Å). Yan et al. also crystallized (PLS)_3_PPR in complex with MORF9, a member of 10 co-factor proteins essential for RNA editing in *A. thaliana* (Bentolila et al. 2012, Takenaka et al. 2012). They found that MORF9 associates with L-motifs through a hydrogen bond between K29 of the L-type PPR motif and D164 of MORF9 and that the interaction of MORF9 with L-type PPR motifs causes an inward rotation of L-type motifs by ∼6°, resulting in the last aa residue of the L-motif being positioned closer to its associated RNA base. Each structure folded into the expected α-solenoid structure of stacked helix-turn-helix motifs forming a superhelical structure with an internal RNA-binding groove.

The ‘dPPR’ synthetic P-type PPR protein scaffold designed by Shen et al. (**Fig. 2**) has been most widely utilized in studies on the binding specificity of synthetic P-type PPR proteins in vivo and in vitro (Miranda et al. 2018, Yan et al. 2019). A synthetic S-type consensus PPR motif dsnSc was designed by Bernath-Levin et al. (2021) to analyze the binding specificities of S-type PPR motifs. The consensus sequence of dsnSc is included in **Fig. 2**, while the design of these motifs is described in more detail later.

### Synthetic PPR proteins used to elucidate details of RNA recognition

Synthetic PPR proteins have provided important insights into PPR protein architecture and the mode of RNA recognition (Coquille et al. 2014, Gully et al. 2015a, Shen et al. 2016), and they were instrumental in developing our understanding of the ways that PPR motifs interact with RNA bases. The success of designing synthetic PPR motifs and heterologously expressing them in *E. coli* allowed for interrogation of the interactions of the fifth and last aas and RNA. Shen et al. (2015) described an in vitro assay using a set of synthetic PPR proteins with PPR10-derived N- and C-terminal caps, which bound radioactively labeled single-stranded RNA using combinations SN → A, ND → U and NS → C (Shen et al. 2015). Shen et al. used their in vitro PPR assay to model the structural basis for RNA recognition in their synthetic PPR protein (Shen et al. 2016). Shen et al. (2016) highlighted the importance of additional aas within the PPR motif contributing to RNA specificity. They showed that the second aa, in particular, contributes to RNA binding. aa 2 in the PPR motif clamps its corresponding nucleobase in a ‘sandwich-like’ manner through van der Waals interactions. Shen et al. also highlighted the importance of aa 13, which is positioned at the extremity of helix A in each repeat. A lysine residue at position 13 contributes to positive electrostatic potential in a PPR motif, facilitating interactions with the negatively charged phosphate group of the RNA base. The K13 phosphate group interactions were present in PPR repeats 1–8 and were mediated by salt bridges. Substitution of K13 with alanine abolished RNA binding completely (Shen et al. 2016). In other synthetic PPR motifs, the aa at position 13 has been substituted with arginine or glutamine, which is compatible with RNA binding (Yan et al. 2019, Bernath-Levin et al. 2021). Polar aas at the fifth position in the PPR motif are one of the major determinants of RNA base specificity, with serine or threonine preferring purines and asparagine preferring pyrimidines. dPPR structures in the study by Shen et al. provided an explanation for the PPR code, showing that an N5 side chain donated a hydrogen bond to the O2 atom of a pyrimidine and the N3 atom of a purine accepts a hydrogen bond from the hydroxyl group of the corresponding aa (e.g. T5 or S5). The 35th aa of the PPR motif is in close proximity to the nucleobase, and Shen et al. (2016) observed that water molecules form hydrogen bonds with the N3 atom of a pyrimidine and the carboxyl group of N35, or a hydroxyl group of S35, showing that base selectivity between aa 35 is determined by ‘water bridge’ polarity. The N3 atom of a uracil is a hydrogen bond donor, whereas the N3 atom of a cytosine is a hydrogen bond acceptor. For purines, N35 or D35 forms one or two hydrogen bonds with adenine and guanine, respectively. The N1 atom of adenine is a hydrogen bond acceptor, whereas N1 and N2 atoms of guanine are hydrogen bond donors.

The initial cohort of all synthetic PPR proteins used a very restricted subset of the possible aa combinations in the PPR code. Subsequent work with synthetic PPR proteins has greatly expanded the experimental data available for understanding PPR binding specificity. In a large-scale study of PPR-RNA-binding affinity, Yan et al. designed a 10-PPR motif P-type PPR protein based on the dsnPPR scaffold in the study by Shen et al.. They then changed the fifth and last aas on two of the 10 PPR motifs and measured the binding affinity of the altered proteins using isothermal titration calorimetry, effectively profiling many combinations of fifth and last aa against each RNA base (Yan et al. 2019). Bernath-Levin et al. (2021) used a less quantitative but high-throughput RNA pull-down approach to profile the binding specificities of even more variants of synthetic S-type PPR motifs (Bernath-Levin et al. 2021). The binding specificities of synthetic S-type motifs were in general similar to those of synthetic P-type motifs, although some differences in strength of the binding of particular fifth/last aa combinations to their preferred RNA base were identified (Bernath-Levin et al. 2021). Combinations including small side chain aas such as serine, glycine and alanine at position 5, which are found to be strongly specific for purines in synthetic P-class proteins, bind weakly and less specifically in the synthetic S-class proteins. Interestingly, these combinations are also much rarer in natural S-class proteins (Bernath-Levin et al. 2021).

The PPR code has been used to guide modification of natural PPR proteins in order to influence their RNA binding specificity or re-target them to different transcripts. Sometimes, this was done to achieve a specific applied outcome, and these examples will be discussed later. However, many of these experiments were basic research aimed at testing the PPR code or deepening our understanding of PPR-RNA recognition. The earliest experiments of this type were the deliberate modifications of PPR10 used to validate the original PPR code (Barkan et al. 2012), where it was shown that altering two motifs in the protein altered its RNA target preference in vitro exactly as predicted. Similar experiments were done on the RNA editing factors CLB19 and OTP82, but this time in vivo (Kindgren et al. 2015). Both PPRs recognize two similar target sites, but could be rendered more or less specific to one or other site by deliberate alterations to relevant PPR motifs (Kindgren et al. 2015). More recently, extensive analyses of PPR binding specificity have relied on the propensity for over-expressed RNA editing factors (natural or synthetic) to catalyze often substantial numbers of off-target events (Oldenkott et al. 2019, Royan et al. 2021, Lesch et al. 2022, Loiacono et al. 2022, Yang et al. 2023). For example, a study using *P. patens* PPR56 and PPR65 heterologously expressed in *E. coli*, HeLa, IMR-90 and HEK-293 cells studied the effects of mutating fifth and last aas of the two PPR proteins on background editing in bacterial and mammalian transcriptomes (Lesch et al. 2022). An EYFP-tagged PPR56 protein expressed in HeLa cells had over 900 off-target RNA editing sites. Many of these off-target sites showed expected nucleotide preferences at sites aligning with P- and S-type motifs, with a preference for pyrimidines opposite L-type motifs. Lesch et al. mutated two S-type motifs (S4TD → TN and S7TN → TD) and noted a significant shift in the profile of off-target editing sites in both mammalian and bacterial transcriptomes. Modifications to two S-type motifs showed expected shifts in purine preference, but interestingly also affected the preference of adjacent L- and P-type motifs for their respective nucleotides, providing the first clear evidence that the current assumption that the specificity of each PPR motif can be considered independent of its neighbors is overly simplistic.

Further investigations into RNA binding by synthetic PPR proteins have revealed other factors influencing PPR-RNA binding, which are key considerations in the design of synthetic PPR proteins. A study of binding affinity using synthetic consensus PPR motif scaffolds of varying lengths to bind a library of randomized RNA sequences determined that the optimal number of motifs for a synthetic P-class PPR protein is ∼11 motifs. Increasing the number of motifs to 14 did not increase specificity; rather, it was observed that the synthetic proteins became more tolerant of mismatches and therefore less specific for their designed RNA targets (Miranda et al. 2018).

### Applications of synthetic PPR proteins: target-specific transcript stabilization and translational activation

**Fig. 3:**
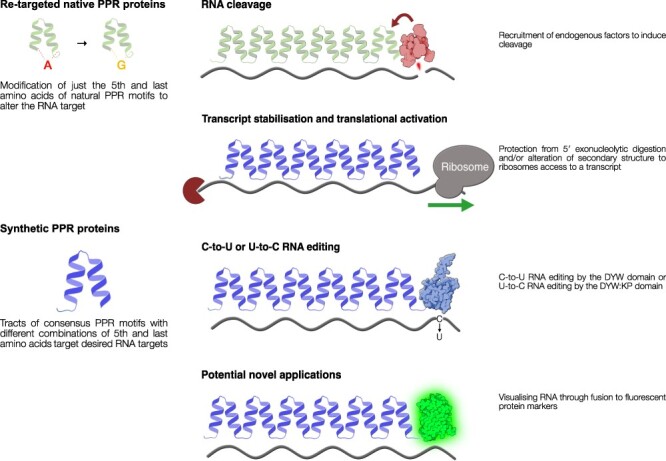
Example use cases for modified and synthetic PPR proteins. Uses of modified natural PPR proteins include using retargeted RFL proteins such as RPF2 (Colas Des Francs-small et al. 2018) to recruit endogenous endonuclease complexes to target RNAs for degradation. Synthetic P-class PPR proteins have been designed to stabilize transcripts by protecting them from 5′ or 3′ exonucleases. They may also be designed to alter the RNA secondary structure to give access to ribosomes, thereby promoting translation of a target transcript. Synthetic PLS-class PPR protein RNA editing factors have been designed to induce single-nucleotide C-to-U transitions via a DYW domain (Royan et al. 2021) or U-to-C transitions via a DYW:KP domain (Ichinose et al. 2022). An array of PPR motifs may be fused to other types of protein domains to confer novel utility such as a fusion to GFP to visualize the localization of target transcripts. Protein domain illustrations were made using *Illustrate* (Goodsell et al. 2019) and the Protein Data Bank (PDB) ID:7W86, PDB ID:4OGS and PDB ID:2MX0.

So far, we have reviewed the use of synthetic PPR proteins in improving our understanding of PPR protein structures and functions. This understanding has advanced sufficiently for a new generation of synthetic PPR proteins to be designed to achieve specific biotechnological goals (see **Fig. 3** for some examples of how such proteins are designed). These goals are often inspired by the roles of natural PPR proteins in different aspects of organelle gene expression and, e.g. include switching the expression of organelle genes ‘on’ or ‘off’, depending on the needs of the application. Many natural PPR proteins act as RNA stabilization factors (Pfalz et al. 2009, Boulouis et al. 2011, Ruwe and Schmitz-Linneweber 2012, Zhelyazkova et al. 2012, Haïli et al. 2013, Wu et al. 2016, Lee et al. 2017, Wang et al. 2017, 2022, Best et al. 2023) and thus act to promote gene expression by increasing the half-life of their target transcripts. The inspiration to use such PPRs as biotechnological tools came from studies demonstrating that the *Chlamydomonas reinhardtii* chloroplast protein Nac2, which naturally stabilizes the *psbD* mRNA (Boudreau et al. 2000), can be used to effectively control the expression of chloroplast transgenes (Surzycki et al. 2007, Rochaix et al. 2021). Nac2 is neither a PPR protein (although similar in structure), nor synthetic (by the definition used in this review), but illustrates one way in which PPR proteins could be used to switch on expression of specific organelle transcripts.

The advantage of using synthetic over natural proteins as regulators of transgene expression is that they can be designed to be ‘orthogonal’ to the endogenous regulatory machinery, i.e. the synthetic protein does not interact with any regulatory elements in endogenous mRNAs, and its target site in the transgene mRNA is not bound by any endogenous regulatory proteins. The potential of this approach has been emphatically demonstrated using a synthetic version of PPR10 originally used to validate the PPR code (Barkan et al. 2012). Deliberate modifications to the fifth and sixth motifs in PPR10 gave synthetic versions that could no longer recognize the original binding site, but that bound avidly to variants with the appropriate nucleotides at the aligned positions. Rojas et al. (2019) used these modified binding sites as activator elements 5ʹ of a transgene in tobacco chloroplasts, resulting in a remarkable up to 40-fold increase in expression of a transgene-encoded reporter protein in the presence of the corresponding synthetic PPR10 variant. The effectiveness of PPR10 in this role may be explained by the fact that not only does it stabilize the target mRNA but also it contributes significantly to activating its translation (Prikryl et al. 2011). The same transgene regulatory system was introduced into potato (*Solanum tuberosum*), where plastid reporter protein increased from 0.06% to 1.2% in tubers by expressing the engineered PPR10 from the tuber-active patatin promoter (Yu et al. 2019). However, this system did not provide complete tissue-specificity, as low levels of reporter protein were still produced in leaves where the engineered PPR protein was not expressed. These examples illustrate that using synthetic variants of native PPR proteins is a viable path to creating novel regulatory systems for organelle transgenes. However, the binding specificities of natural PPRs are often influenced by non-canonical interactions that are not currently described by the PPR code. This makes re-targeting natural PPR proteins somewhat unpredictable and time consuming, as well as limiting the number of possible modifications.

More options would be available if fully synthetic PPR proteins could be used in the same way. Manavski et al. (2021) took the first steps toward this goal by demonstrating the potential of fully synthetic PPR proteins to stabilize chloroplast mRNAs in vivo. Two synthetic P-type PPR proteins were targeted to the binding sites of natural *Arabidopsis* P-type PPR proteins MRL1 and PGR3, and they effectively stabilized the 5ʹ ends of target transcripts in *Arabidopsis* mutants lacking the corresponding natural PPR protein. However, transcript stabilization by the synthetic PPR proteins only resulted in minor improvements in target protein synthesis. Therefore, the potential of this strategy for biotechnology requires further exploration.

### Applications of synthetic PPR proteins: target-specific RNA cleavage

PPR proteins can also be used to turn off organelle gene expression. Restorer-of-fertility PPR proteins act to suppress expression of mitochondrial genes that cause cytoplasmic male sterility (CMS), generally by binding to the CMS-causing mRNA and inducing its cleavage (Kazama et al. 2008, Melonek et al. 2021) or blocking its translation (Wang et al. 2021). This expression suppression effect can be manipulated by re-targeting the PPR to a different transcript. This has now been done twice with synthetic variants of the *Arabidopsis* RFL protein RPF2, whose native targets are the 5ʹ UTRs of the mitochondrial transcripts *cox3* and *nad9*. RPF2 was redesigned to target the *nad6* or *atp1* mRNAs within their coding sequences (Colas Des Francs-small et al. 2018, Yang et al. 2022). In both cases, the synthetic PPRs induced cleavage of the target mRNAs and thus the reduction of the Nad6 and Atp1, respectively, resulting in low levels of assembled complexes I and V. Very few off-target binding events were detected (Colas Des Francs-small et al. 2018, Yang et al. 2022). The potential applications of this approach are relatively limited, though, as the re-targeting of natural PPR proteins tends to be only successful for sites very similar to the original target site, and the RNA cleavage induced by these proteins is almost certainly dependent on an endogenous endonuclease (Huynh et al. 2023), making it unlikely that these proteins would induce cleavage in other genetic systems, even chloroplasts.

An ideal solution would be to combine the RNA-binding PPR array and the endonuclease activity in a single protein. This combination does occur naturally, the best-studied examples being proteinaceous RNase P (PRORP) proteins. PRORP1 is an organelle-targeted RNase P enzyme that endonucleolytically cleaves tRNA precursors at the 5ʹ end of the mature tRNA (Gobert et al. 2010). PRORP proteins contain 2–3 PPR motifs that bind RNA, with endonucleolytic cleavage catalyzed by a C-terminal YacP nuclease domain. Gobert et al. removed the nuclear localization sequence from PRORP2 and demonstrated its activity to cleave viral tRNA-like structures commonly present in plant viral transcripts (Gobert et al. 2021). However, whether synthetic versions of PRORP proteins that could target any RNA sequence could be constructed is still unknown. A second potential route to a generic synthetic PPR-endonuclease is via PPR-SMR proteins. The SMR domain is found in a select group of P-class PPRs in plants (Liu et al. 2013) and has been associated with endonucleolytic activity (Zhou et al. 2017). At least in vitro, the PPR-SMR protein SOT1 can be engineered to target and cleave alternative RNA sequences, but as for other engineered variants of natural PPR proteins, it is likely that there is a limited scope for targeting a wide range of different sequences. As yet, the successful combination of a fully synthetic PPR array capable of being designed to target any sequence together with an effective RNA endonuclease domain has not been reported.

### Applications of synthetic PPR proteins: target-specific RNA editing

Much more precise and subtle control of gene expression could be achieved by altering the sequence of the target RNA rather than simply stabilizing or destabilizing it. Using synthetic RNA editing factors to selectively alter transcripts is thus a long-term goal of synthetic PPR protein research. The first fully synthetic C-to-U RNA editing factors were designed and tested in *E. coli* and *A. thaliana* using scaffolds of PLS motifs (Royan et al. 2021) and S-type motifs (Bernath-Levin et al. 2021). Royan et al. designed a novel synthetic PPR protein with the motif arrangement (P1–L1–S1)_3_–P2–L2–S2–E1–E2–DYW based on representative aas at each position in each motif based on 9730 PPR protein sequences from 38 species of seed and non-seed plants. The synthetic PPR protein, called ‘dsn3PLS-DYW’, was targeted to bind the RNA sequence upstream of the *A. thaliana* chloroplast *rpoA-78691* C-to-U RNA editing site. The *rpoA-78691* editing site in the *A. thaliana* chloroplast transcriptome is one of two RNA editing sites targeted by the PLS-class PPR protein CLB19, with the other being *clpP1-69942* (Chateigner-Boutin et al. 2008). The dsn3PLS-DYW synthetic PPR protein was designed to selectively edit just the *rpoA-78691* site. It was able to edit its target transcript up to 37% in bacteria in combination with the co-factor protein MORF9 and ∼40% in planta in the presence of MORF9. In the absence of MORF co-factors, dsn3PLS-DYW was able to edit just ∼8% of its target transcript. A synthetic RNA editing factor utilizing an array of synthetic S-type PPR motifs, designed largely based on the S-type PPR-RNA editing factors of seed-free plants, was also demonstrated to edit RNA in bacteria (Bernath-Levin et al. 2021). A nine-motif synthetic S-type PPR protein with a C-terminal P2–L2–S2–E1–E2–DYW RNA editing domain was assembled targeting the *rpoA-78691* target of the *A. thaliana rpoA* transcript. When expressed in *E. coli*, TRX-9S-DYW was able to bind and edit the *rpoA* target site and edit *rpoA-78691* without reliance on the MORF co-factor proteins that were required by dsn3PLS-DYW (Bernath-Levin et al. 2021, Royan et al. 2021). This co-factor-independent synthetic protein achieved up to ∼50% conversion of cytidine to uridine in the presence or absence of MORF2 (Bernath-Levin et al. 2021).

Ichinose et al. were the first to show site-specific U-to-C RNA editing by synthetic PPR protein in *E. coli* and human cell cultures, while also experimentally validating that U-to-C RNA editing is carried out by PPR proteins with the ‘KP’ variant of the DYW domain (Ichinose et al. 2022). In this study, a synthetic PLS-type (P1–L1–S1)_3_ PPR tract was designed based on consensus sequences of PPR motifs from 66 plant genomes. As in the study by Royan et al. and Bernath-Levin et al., the (P1–L1–S1)_3_ tract was targeted to bind sequences upstream of the *rpoA* editing site. The same PPR tract was fused to seven different C-terminal sequences, each encoding for P2–L2–S2–E1–E2–DYW:KP motifs designed based on consensus sequences of these motifs from PPR proteins previously suggested to carry out U-to-C editing in seed-free plants (Gerke et al. 2020). Three of the resulting designer proteins were functional and achieved editing efficiencies of up to 50% in *E. coli* and 28% in HEK293T human cells (Ichinose et al. 2022). One of the three proteins was also observed to have low levels of C-to-U editing activity in HEK293T cells. Interestingly, the presence of MORF2 or MORF9 proteins did not improve editing efficiency of the proteins even though the (P1–L1–S1)_3_ PPR tract in this study had 95% identity with the (P1–L1–S1)_3_ sequence in Royan et al. (2021).

Research on synthetic RNA editing factors is looking promising, but as yet, there are no published demonstrations that a synthetic RNA editing factor can be designed to target a completely novel editing site, the publications to date either target known editing sites or report off-target events at novel sites that were not the intended target. The ability to target any desired site would open up some exciting possibilities for controlling gene expression in new ways, e.g. by the creation of start or stop codons (via C-to-U editing) or by their removal (via U-to-C editing).

### Applications of synthetic PPR proteins: novel and potential applications

We have covered the major areas of basic and applied research on synthetic PPR proteins, but many other potential uses are being explored or can be envisaged. In addition to designing synthetic PPR proteins that substitute for functions carried out by natural PPR proteins, such as RNA editing or transcript stabilization, synthetic PPR proteins have been engineered for entirely novel uses. For example, many natural PPR proteins are implicated in RNA splicing, but how they act in these processes is too uncertain for the time being to engineer PPR proteins to predictably influence plant organellar RNA splicing. However, PPR proteins can be used to control alternative splicing in mammalian cells by deliberately targeting the PPRs at sequences required for exon recognition (Yagi et al. 2022). In this study, the authors used synthetic PPR proteins to promote exon-skipping in transcripts encoding a bi-chromatic fluorescent reporter protein in HEK293T cells. They went on to demonstrate that the same approach could work to influence exon-skipping of endogenous mRNAs in the same cells (Yagi et al. 2022). This is exploiting the sequence-specific RNA binding ability of PPR proteins to disrupt a process that they are not naturally involved in (exon recognition in the mammalian cytosol and plant organelles differs greatly).

Tight sequence–specific binding by PPR proteins can be exploited in other ways. McDermott et al. demonstrated the use of synthetic PPR proteins as a research tool to identify proteins that bind specific RNA sequences in vivo. They generated stably transformed *Arabidopsis* plants expressing 3× FLAG-tagged synthetic 11 and 14 motif P-type PPR proteins designed to bind the 3ʹ untranslated region of chloroplast *psbA* mRNA. They first verified the binding of the proteins to the intended target RNA sequence in vivo by co-immunoprecipitation sequencing (RIP-seq) (McDermott et al. 2019). They then identified other proteins that interact with the *psbA* mRNA by detecting proteins that were present in the RIP-seq co-immunoprecipitates using mass spectrometry. This novel use of synthetic PPR proteins for RNA capture could be widely used in plant organelles to identify proteins that interact with a specific RNA of interest.

Finally, synthetic PPR proteins are not limited to applications involving RNA. A synthetic PPR protein has been designed to bind single-stranded telomeric DNA (Spåhr et al. 2018). The bound PPR protein inhibited human telomerase activity (Spåhr et al. 2018). The specificity of DNA binding was guided by the same PPR code as for RNA, so other single-stranded DNA targets can potentially be targeted.

### Molecular cloning strategies for synthetic PPR proteins

Repetitive DNA sequences can be a significant challenge for traditional molecular cloning techniques. DNA sequences encoding for native PPR proteins tend to be relatively long and highly repetitive, which makes them difficult to modify and clone by PCR-based techniques (Hommelsheim et al. 2014). This is an even more acute issue for genes encoding for fully synthetic PPR proteins that consist of short repetitive sequences with variation only at certain codons. Repetitive sequences may suffer from unwanted recombination when using DNA assembly methods that rely on homologous recombination between adjacent DNA fragments, such as Gibson assembly. Genes encoding for synthetic PPR proteins can be synthesized de novo either in whole or in part [e.g. as in Royan et al. (2021)]. However, DNA synthesis companies often charge a premium for cloning long repetitive sequences or may reject the gene synthesis order altogether. A more cost-effective approach is to order repetitive DNA sequences synthesized in multiple shorter 300- to 500-bp blocks, and to assemble them using a modular cloning system based on type IIS restriction enzymes, such as the bacterial and plant MoClo system (Weber et al. 2011) or loop assembly (Pollak et al. 2019).

Two groups have developed rapid modular assembly systems to construct libraries of synthetic PPR proteins (Yan et al. 2019, Yagi et al. 2022). However, the requirement to maintain the sequence identity of the last aa of the PPR motif makes the design of a modular library of PPR gene fragments challenging. Yan et al. created a set of PPR monomers by overlapping PCR using long primers with unique linkers, specifying the position for each monomer in a final assembly reaction. Monomers were assembled into 3-mers of PPR motifs, which were assembled into a final vector encoding a 10-repeat designer P-class PPR protein (Yan et al. 2019). Yagi et al. employed the principles of Golden Gate assembly to construct a library of 2-mer PPR repeats as a set of 144 plasmids to assemble P-class PPR proteins with 18 motifs for expression in *E. coli* (Yagi et al. 2022). With this library of modular DNA components, higher-throughput experiments are possible as new synthetic PPR proteins can be assembled to modify target specificity by altering which parts are used in the DNA assembly reaction. Widely distributed libraries of PPR motif modules will be essential for the ultimate goal of being able to rapidly design and implement synthetic PPR proteins for use in targeted RNA binding and RNA editing.

## Perspectives for Synthetic PPR Proteins

Synthetic PPR proteins have the potential to become powerful RNA processing tools with applications in agriculture, biotechnology and medicine, particularly when organellar RNA is the target. The most obvious potential uses of synthetic PPRs have been demonstrated, at least in principle, in a few specific cases. Progress has been particularly rapid over the last 2–3 years. However, what is still lacking is a widely available modular cloning system for user-friendly construction of custom PPR sequences to target any RNA and indeed the demonstration that a large fraction of synthetic PPRs bind their intended target. We still have a limited understanding of the ways that PPR proteins holistically interact with RNA, that is, how they recognize RNA molecules beyond the interactions of RNA with two critical aas at the fifth and last positions in each PPR motif, and thus, we may find that a significant fraction of synthetic PPRs do not perform as expected. Finally, even when these issues are solved, there remain questions about the specificity of synthetic PPRs in complex transcriptomes. As discussed in McDowell et al. (2022), for applications in eukaryotic cytosolic or nuclear compartments, it may be necessary to use split-effector approaches that rely on binding of two different PPR proteins to achieve the requisite binding specificity.

## Data Availability

No new datasets were generated or analyzed in this study.

## References

[R1] Andrés-Colás N., Zhu Q., Takenaka M., De Rybel B., Weijers D. and Van Der Straeten D. (2017) Multiple PPR protein interactions are involved in the RNA editing system in Arabidopsis mitochondria and plastids. *Proc. Natl. Acad. Sci. U.S.A.* 114: 8883–8888. 28761003 10.1073/pnas.1705815114 PMC5565447

[R2] Aryamanesh N., Ruwe H., Sanglard L.V.P., Eshraghi L., Bussell J.D., Howell K.A., et al. (2017) The pentatricopeptide repeat protein EMB2654 is essential for trans-splicing of a chloroplast small ribosomal subunit transcript. *Plant Physiol.* 173: 1164–1176. 28011633 10.1104/pp.16.01840 PMC5291019

[R3] Barkan A., Rojas M., Fujii S., Yap A., Chong Y.S., Bond C.S., et al. (2012) A combinatorial amino acid code for RNA recognition by pentatricopeptide repeat proteins. *PLoS Genet.* 8: e1002910. 10.1371/journal.pgen.1002910 PMC3420917 22916040

[R4] Barkan A. and Small I. (2014) Pentatricopeptide repeat proteins in plants. *Annu. Rev. Plant Biol.* 65: 415–442. 24471833 10.1146/annurev-arplant-050213-040159

[R5] Beick S., Schmitz-Linneweber C., Williams-Carrier R., Jensen B. and Barkan A. (2008) The pentatricopeptide repeat protein PPR5 stabilizes a specific tRNA precursor in maize chloroplasts. *Mol. Cell Biol.* 28: 5337–5347. 18591259 10.1128/MCB.00563-08 PMC2519714

[R6] Bentolila S., Heller W.P., Sun T., Babina A.M., Friso G., Van Wijk K.J., et al. (2012) RIP1, a member of an Arabidopsis protein family, interacts with the protein RARE1 and broadly affects RNA editing. *Proc. Natl. Acad. Sci. U.S.A.* 109: E1453–E1461. 22566615 10.1073/pnas.1121465109 PMC3365174

[R7] Bernath-Levin K., Schmidberger J., Honkanen S., Gutmann B., Sun Y.K., Pullakhandam A., et al. (2021) Cofactor-independent RNA editing by a synthetic S-type PPR protein. *Synth. Biol.* 7: ysab034. 10.1093/synbio/ysab034 PMC8809517 35128071

[R8] Best C., Mizrahi R., Edris R., Tang H., Zer H., Colas Des Francs-small C., et al. (2023) MSP1 encodes an essential RNA-binding pentatricopeptide repeat factor required for nad1 maturation and complex I biogenesis in Arabidopsis mitochondria. *New Phytologist* 238: 2375–2392. 36922396 10.1111/nph.18880

[R9] Binder S., Stoll K. and Stoll B. (2013) P-class pentatricopeptide repeat proteins are required for efficient 5ʹ end formation of plant mitochondrial transcripts. *RNA Biol.* 10: 1511–1519. 24184847 10.4161/rna.26129 PMC3858434

[R10] Boudreau E., Nickelsen J., Lemaire S.D., Ossenbühl F. and Rochaix J.D. (2000) The Nac2 gene of Chlamydomonas encodes a chloroplast TPR-like protein involved in *psbD* mRNA stability. *EMBO J.* 19: 3366–3376. 10880449 10.1093/emboj/19.13.3366 PMC313939

[R11] Boulouis A., Raynaud C., Bujaldon S., Aznar A., Wollman F.-A. and Choquet Y. (2011) The nucleus-encoded trans-acting factor MCA1 plays a critical role in the regulation of cytochrome f synthesis in Chlamydomonas chloroplasts. *Plant Cell* 23: 333–349. 21216944 10.1105/tpc.110.078170 PMC3051260

[R12] Boussardon C., Salone V., Avon A., Berthomé R., Hammani K., Okuda K., et al. (2012) Two interacting proteins are necessary for the editing of the NdhD-1 site in Arabidopsis plastids. *Plant Cell* 24: 3684–3694. 23001034 10.1105/tpc.112.099507 PMC3480295

[R13] Burjoski V. and Reddy A.S.N. (2021) The landscape of RNA-protein interactions in plants: approaches and current status. *Int. J. Mol. Sci.* 22: 2845. 10.3390/ijms22062845 PMC7999938 33799602

[R14] Chateigner-Boutin A.-L., Colas Des Francs-small C., Delannoy E., Kahlau S., Tanz S.K., Falcon de Longevialle A., et al. (2011) OTP70 is a pentatricopeptide repeat protein of the E subgroup involved in splicing of the plastid transcript rpoC1. *Plant J.* 65: 532–542. 21288264 10.1111/j.1365-313X.2010.04441.x

[R15] Chateigner-Boutin A.-L., Ramos-Vega M., Guevara-García A., Andrés C., de la Luz Gutiérrez-nava M., Cantero A., et al. (2008) CLB19, a pentatricopeptide repeat protein required for editing of rpoA and clpP chloroplast transcripts. *Plant J.* 56: 590–602. 18657233 10.1111/j.1365-313X.2008.03634.x

[R16] Cheng S., Gutmann B., Zhong X., Ye Y., Fisher M.F., Bai F., et al. (2016) Redefining the structural motifs that determine RNA binding and RNA editing by pentatricopeptide repeat proteins in land plants. *Plant J.* 85: 532–547. 26764122 10.1111/tpj.13121

[R17] Colas Des Francs-small C., Vincis Pereira Sanglard L. and Small I. (2018) Targeted cleavage of nad6 mRNA induced by a modified pentatricopeptide repeat protein in plant mitochondria. *Commun. Biol.* 1: 166. 10.1038/s42003-018-0166-8 PMC6181959 30320233

[R18] Coquille S., Filipovska A., Chia T., Rajappa L., Lingford J.P., Razif M.F.M., et al. (2014) An artificial PPR scaffold for programmable RNA recognition. *Nat. Commun.* 5: 1–9. 10.1038/ncomms6729 25517350

[R19] Dasgupta S. and Bell J.A. (1993) Design of helix ends. Amino acid preferences, hydrogen bonding and electrostatic interactions. *Int. J. Pept. Protein Res.* 41: 499–511. 8320043

[R20] Falcon de Longevialle A., Meyer E.H., Andrés C., Taylor N.L., Lurin C., Millar A.H., et al. (2007) The pentatricopeptide repeat gene OTP43 is required for trans-splicing of the mitochondrial nad1 Intron 1 in Arabidopsis thaliana. *Plant Cell* 19: 3256–3265. 17965268 10.1105/tpc.107.054841 PMC2174710

[R21] Filipovska A., Razif M.F.M., Nygård K.K.A. and Rackham O. (2011) A universal code for RNA recognition by PUF proteins. *Nat. Chem. Biol.* 7: 425–427. 21572425 10.1038/nchembio.577

[R22] Fujii S., Bond C.S. and Small I.D. (2011) Selection patterns on restorer-like genes reveal a conflict between nuclear and mitochondrial genomes throughout angiosperm evolution. *Proc. Natl. Acad. Sci. U.S.A.* 108: 1723–1728. 21220331 10.1073/pnas.1007667108 PMC3029733

[R23] Gerke P., Szövényi P., Neubauer A., Lenz H., Gutmann B., McDowell R., et al. (2020) Towards a plant model for enigmatic U-to-C RNA editing: the organelle genomes, transcriptomes, editomes and candidate RNA editing factors in the hornwort Anthoceros agrestis. *New Phytologist* 225: 1974–1992. 31667843 10.1111/nph.16297

[R24] Gobert A., Gutmann B., Taschner A., Gössringer M., Holzmann J., Hartmann R.K., et al. (2010) A single Arabidopsis organellar protein has RNase P activity. *Nat. Struct. Mol. Biol.* 17: 740–744. 20473316 10.1038/nsmb.1812

[R25] Gobert A., Quan Y., Arrivé M., Waltz F., Da Silva N., Jomat L., et al. (2021) Towards plant resistance to viruses using protein-only RNase P. *Nat. Commun.* 12: 1007. 10.1038/s41467-021-21338-6 PMC7881203 33579946

[R26] Goodsell D.S., Autin L. and Olson A.J. (2019) Illustrate: software for biomolecular illustration. *Structure* 27: 1716–1720.e1. 31519398 10.1016/j.str.2019.08.011 PMC6834899

[R27] Guillaumot D., Lopez-Obando M., Baudry K., Avon A., Rigaill G., Falcon de Longevialle A., et al. (2017) Two interacting PPR proteins are major Arabidopsis editing factors in plastid and mitochondria. *Proc. Natl. Acad. Sci. U.S.A.* 114: 8877–8882. 28760958 10.1073/pnas.1705780114 PMC5565446

[R28] Gully B.S., Cowieson N., Stanley W.A., Shearston K., Small I.D., Barkan A., et al. (2015a) The solution structure of the pentatricopeptide repeat protein PPR10 upon binding atpH RNA. *Nucleic Acids Res.* 43: 1918–1926. 25609698 10.1093/nar/gkv027 PMC4330388

[R29] Gully B.S., Shah K.R., Lee M., Shearston K., Smith N.M., Sadowska A., et al. (2015b) The design and structural characterization of a synthetic pentatricopeptide repeat protein. *Acta Crystallogr. D Biol. Crystallogr.* 71: 196–208. 25664731 10.1107/S1399004714024869

[R30] Gutmann B., Royan S., Schallenberg-Rüdinger M., Lenz H., Castleden I.R., McDowell R., et al. (2020) The expansion and diversification of pentatricopeptide repeat RNA-editing factors in plants. *Mol. Plant* 13: 215–230. 31760160 10.1016/j.molp.2019.11.002

[R31] Haïli N., Arnal N., Quadrado M., Amiar S., Tcherkez G., Dahan J., et al. (2013) The pentatricopeptide repeat MTSF1 protein stabilizes the *nad4* mRNA in *Arabidopsis* mitochondria. *Nucleic Acids Res.* 41: 6650–6663. 23658225 10.1093/nar/gkt337 PMC3711453

[R32] Harrison T., Ruiz J., Sloan D.B., Ben-Hur A., Boucher C. and Promponas V.J. (2016) aPPRove: an HMM-based method for accurate prediction of RNA-pentatricopeptide repeat protein binding events. *PLoS One* 11: e0160645. 10.1371/journal.pone.0160645 PMC4999063 27560805

[R33] Hayes M.L. and Santibanez P.I. (2020) A plant pentatricopeptide repeat protein with a DYW-deaminase domain is sufficient for catalyzing C-to-U RNA editing in vitro. *J. Biol. Chem.* 295: 3497–3505. 31996373 10.1074/jbc.RA119.011790 PMC7076202

[R34] Hommelsheim C.M., Frantzeskakis L., Huang M. and Ülker B. (2014) PCR amplification of repetitive DNA: a limitation to genome editing technologies and many other applications. *Sci. Rep.* 4: 5052. 10.1038/srep05052 PMC4031481 24852006

[R35] Howard M.J., Lim W.H., Fierke C.A. and Koutmos M. (2012) Mitochondrial ribonuclease P structure provides insight into the evolution of catalytic strategies for precursor-tRNA 5ʹ processing. *Proc. Natl. Acad. Sci. U.S.A.* 109: 16149–16154. 22991464 10.1073/pnas.1209062109 PMC3479547

[R36] Huynh S.D., Melonek J., Colas Des Francs-small C., Bond C.S. and Small I. (2023) A unique C-terminal domain contributes to the molecular function of Restorer-of-fertility proteins in plant mitochondria. *New Phytologist* 240: 830–845. 37551058 10.1111/nph.19166

[R37] Ichinose M., Kawabata M., Akaiwa Y., Shimajiri Y., Nakamura I., Tamai T., et al. (2022) U-to-C RNA editing by synthetic PPR-DYW proteins in bacteria and human culture cells. *Commun. Biol.* 5: 968. 10.1038/s42003-022-03927-3 PMC9478123 36109586

[R38] Kajander T., Cortajarena A.L., Mochrie S. and Regan L. (2007) Structure and stability of designed TPR protein superhelices: unusual crystal packing and implications for natural TPR proteins. *Acta Crystallogr. D Biol. Crystallogr.* 63: 800–811. 17582171 10.1107/S0907444907024353

[R39] Kajander T., Cortajarena A.L. and Regan L. (2006) Consensus design as a tool for engineering repeat proteins. *Methods Mol. Biol.* 340: 151–170. 16957336 10.1385/1-59745-116-9:151

[R40] Kavuri N.R., Ramasamy M., Qi Y. and Mandadi K. (2022) Applications of CRISPR/Cas13-based RNA editing in plants. *Cells* 11: 2665. 10.3390/cells11172665 PMC9454418 36078073

[R41] Kazama T., Nakamura T., Watanabe M., Sugita M. and Toriyama K. (2008) Suppression mechanism of mitochondrial ORF79 accumulation by Rf1 protein in BT-type cytoplasmic male sterile rice. *Plant J.* 55: 619–628. 18435825 10.1111/j.1365-313X.2008.03529.x

[R42] Ke J., Chen R.-Z., Ban T., Zhou X.E., Gu X., Tan M.H.E., et al. (2013) Structural basis for RNA recognition by a dimeric PPR-protein complex. *Nat. Struct. Mol. Biol.* 20: 1377–1382. 24186060 10.1038/nsmb.2710

[R43] Kindgren P., Yap A., Bond C.S. and Small I. (2015) Predictable alteration of sequence recognition by RNA editing factors from Arabidopsis. *Plant Cell* 27: 403–416. 25649437 10.1105/tpc.114.134189 PMC4456925

[R44] Knoop V. and Marquardt S. (2023) C-to-U and U-to-C: RNA editing in plant organelles and beyond. *J. Exp. Bot.* 74: 2273–2294. 36527364 10.1093/jxb/erac488

[R45] Kobayashi T., Yagi Y. and Nakamura T. (2019) Comprehensive prediction of target RNA editing sites for PLS-class PPR proteins in Arabidopsis thaliana. *Plant Cell Physiol.* 60: 862–874. 30605550 10.1093/pcp/pcy251

[R46] Kotera E., Tasaka M. and Shikanai T. (2005) A pentatricopeptide repeat protein is essential for RNA editing in chloroplasts. *Nature* 433: 326–330. 15662426 10.1038/nature03229

[R47] Lee K., Han J.H., Park Y.-I., Colas Des Francs-small C., Small I. and Kang H. (2017) The mitochondrial pentatricopeptide repeat protein PPR19 is involved in the stabilization of NADH dehydrogenase 1 transcripts and is crucial for mitochondrial function and Arabidopsis thaliana development. *New Phytologist* 215: 202–216. 28332713 10.1111/nph.14528

[R48] Lee K., Park S.J., Colas Des Francs-small C., Whitby M., Small I. and Kang H. (2019) The coordinated action of PPR4 and EMB2654 on each intron half mediates trans-splicing of rps12 transcripts in plant chloroplasts. *Plant J.* 100: 1193–1207. 31442349 10.1111/tpj.14509

[R49] Lesch E., Schilling M.T., Brenner S., Yang Y., Gruss O.J., Knoop V., et al. (2022) Plant mitochondrial RNA editing factors can perform targeted C-to-U editing of nuclear transcripts in human cells. *Nucleic Acids Res.* 50: 9966–9983. 36107771 10.1093/nar/gkac752 PMC9508816

[R50] Liu S., Melonek J., Boykin L.M., Small I. and Howell K.A. (2013) PPR-SMRs: ancient proteins with enigmatic functions. *RNA Biol.* 10: 1501–1510. 24004908 10.4161/rna.26172 PMC3858433

[R51] Loiacono F.V., Walther D., Seeger S., Thiele W., Gerlach I., Karcher D., et al. (2022) Emergence of novel RNA-editing sites by changes in the binding affinity of a conserved PPR protein. *Mol. Biol. Evol.* 39: msac222. 10.1093/molbev/msac222 PMC9750133 36227729

[R52] Lurin C., Andrés C., Aubourg S., Bellaoui M., Bitton F., Bruyère C., et al. (2004) Genome-wide analysis of Arabidopsis pentatricopeptide repeat proteins reveals their essential role in organelle biogenesis. *Plant Cell* 16: 2089–2103. 15269332 10.1105/tpc.104.022236 PMC519200

[R53] Main E.R.G., Jackson S.E. and Regan L. (2003a) The folding and design of repeat proteins: reaching a consensus. *Curr. Opin. Struct. Biol.* 13: 482–489. 12948778 10.1016/s0959-440x(03)00105-2

[R54] Main E.R.G., Xiong Y., Cocco M.J., D’Andrea L. and Regan L. (2003b) Design of stable alpha-helical arrays from an idealized TPR motif. *Structure* 11: 497–508. 12737816 10.1016/s0969-2126(03)00076-5

[R55] Manavski N., Mathieu S., Rojas M., Méteignier L.-V., Brachmann A., Barkan A., et al. (2021) In vivo stabilization of endogenous chloroplast RNAs by customized artificial pentatricopeptide repeat proteins. *Nucleic Acids Res.* 49: 5985–5997. 34037778 10.1093/nar/gkab390 PMC8191804

[R56] Marondedze C. (2020) The increasing diversity and complexity of the RNA-binding protein repertoire in plants. *Proc. Biol. Sci.* 287: 20201397. 10.1098/rspb.2020.1397 PMC7542812 32962543

[R57] Marondedze C., Thomas L., Serrano N.L., Lilley K.S. and Gehring C. (2016) The RNA-binding protein repertoire of Arabidopsis thaliana. *Sci. Rep.* 6: 29766. 10.1038/srep29766 PMC4942612 27405932

[R58] McDermott J.J., Watkins K.P., Williams-Carrier R. and Barkan A. (2019) Ribonucleoprotein capture by in vivo expression of a designer pentatricopeptide repeat protein in Arabidopsis. *Plant Cell* 31: 1723–1733. 31123048 10.1105/tpc.19.00177 PMC6713294

[R59] McDowell R., Small I. and Bond C.S. (2022) Synthetic PPR proteins as tools for sequence-specific targeting of RNA. *Methods* 208: 19–26. 36265563 10.1016/j.ymeth.2022.10.003

[R60] Melonek J., Duarte J., Martin J., Beuf L., Murigneux A., Varenne P., et al. (2021) The genetic basis of cytoplasmic male sterility and fertility restoration in wheat. *Nat. Commun.* 12: 1036. 10.1038/s41467-021-21225-0 PMC7884431 33589621

[R61] Miranda R.G., McDermott J.J. and Barkan A. (2018) RNA-binding specificity landscapes of designer pentatricopeptide repeat proteins elucidate principles of PPR-RNA interactions. *Nucleic Acids Res.* 46: 2613–2623. 29294070 10.1093/nar/gkx1288 PMC5861457

[R62] Oldenkott B., Yang Y., Lesch E., Knoop V. and Schallenberg-Rüdinger M. (2019) Plant-type pentatricopeptide repeat proteins with a DYW domain drive C-to-U RNA editing in Escherichia coli. *Commun. Biol.* 2: 85. 10.1038/s42003-019-0328-3 PMC6397227 30854477

[R63] Pfalz J., Bayraktar O.A., Prikryl J. and Barkan A. (2009) Site-specific binding of a PPR protein defines and stabilizes 5′ and 3′ mRNA termini in chloroplasts. *EMBO J.* 28: 2042–2052. 19424177 10.1038/emboj.2009.121 PMC2718276

[R64] Pollak B., Cerda A., Delmans M., Álamos S., Moyano T., West A., et al. (2019) Loop assembly: a simple and open system for recursive fabrication of DNA circuits. *New Phytologist* 222: 628–640. 30521109 10.1111/nph.15625

[R65] Prikryl J., Rojas M., Schuster G. and Barkan A. (2011) Mechanism of RNA stabilization and translational activation by a pentatricopeptide repeat protein. *Proc. Natl. Acad. Sci. U.S.A.* 108: 415–420. 21173259 10.1073/pnas.1012076108 PMC3017144

[R66] Richardson J.S. and Richardson D.C. (1988) Amino acid preferences for specific locations at the ends of alpha helices. *Science* 240: 1648–1652. 3381086 10.1126/science.3381086

[R67] Ringel R., Sologub M., Morozov Y.I., Litonin D., Cramer P. and Temiakov D. (2011) Structure of human mitochondrial RNA polymerase. *Nature* 478: 269–273. 21947009 10.1038/nature10435

[R68] Rivals E., Bruyère C., Toffano-Nioche C. and Lecharny A. (2006) Formation of the Arabidopsis pentatricopeptide repeat family. *Plant Physiol.* 141: 825–839. 16825340 10.1104/pp.106.077826 PMC1489915

[R69] Rochaix J.-D., Surzycki R. and Ramundo S. (2021) Regulated chloroplast gene expression in Chlamydomonas. *Methods Mol. Biol.* 2317: 305–318. 34028778 10.1007/978-1-0716-1472-3_18

[R70] Rojas M., Ruwe H., Miranda R.G., Zoschke R., Hase N., Schmitz-Linneweber C., et al. (2018) Unexpected functional versatility of the pentatricopeptide repeat proteins PGR3, PPR5 and PPR10. *Nucleic Acids Res.* 155: 1520–1512. 10.1093/nar/gky737 PMC6212717 30125002

[R71] Rojas M., Yu Q., Williams-Carrier R., Maliga P. and Barkan A. (2019) Engineered PPR proteins as inducible switches to activate the expression of chloroplast transgenes. *Nat. Plants* 5: 505–511. 31036912 10.1038/s41477-019-0412-1

[R72] Royan S., Gutmann B., Colas Des Francs-small C., Honkanen S., Schmidberger J., Soet A., et al. (2021) A synthetic RNA editing factor edits its target site in chloroplasts and bacteria. *Commun. Biol.* 4: 545. 10.1038/s42003-021-02062-9 PMC8110955 33972654

[R73] Ruwe H. and Schmitz-Linneweber C. (2012) Short non-coding RNA fragments accumulating in chloroplasts: footprints of RNA binding proteins? *Nucleic Acids Res.* 40: 3106–3116. 22139936 10.1093/nar/gkr1138 PMC3326302

[R74] Salone V., Rüdinger M., Polsakiewicz M., Hoffmann B., Groth-Malonek M., Szurek B., et al. (2007) A hypothesis on the identification of the editing enzyme in plant organelles. *FEBS Lett.* 581: 4132–4138. 17707818 10.1016/j.febslet.2007.07.075

[R75] Schallenberg-Rüdinger M. Knoop V. (2016) Coevolution of organelle RNA editing and nuclear specificity factors in early land plants. *In* Genomes and Evolution of Charophytes, Bryophytes, Lycophytes and Ferns, Advances in Botanical Research. Edited by Rensing, S.A. pp. 37–93. Elsevier: Amsterdam.

[R76] Schmitz-Linneweber C., Williams-Carrier R.E., Williams-Voelker P.M., Kroeger T.S., Vichas A. and Barkan A. (2006) A pentatricopeptide repeat protein facilitates the trans-splicing of the maize chloroplast rps12 pre-mRNA. *Plant Cell* 18: 2650–2663. 17041147 10.1105/tpc.106.046110 PMC1626628

[R77] Shen C., Wang X., Liu Y., Li Q., Yang Z., Yan N., et al. (2015) Specific RNA recognition by designer pentatricopeptide repeat protein. *Mol. Plant* 8: 667–670. 25598142 10.1016/j.molp.2015.01.001

[R78] Shen C., Zhang D., Guan Z., Liu Y., Yang Z., Yang Y., et al. (2016) Structural basis for specific single-stranded RNA recognition by designer pentatricopeptide repeat proteins. *Nat. Commun.* 7: 1–8. 10.1038/ncomms11285 PMC4837458 27088764

[R79] Small I.D. and Peeters N. (2000) The PPR motif – a TPR-related motif prevalent in plant organellar proteins. *Trends Biochem. Sci.* 25: 46–47. 10664580 10.1016/s0968-0004(99)01520-0

[R80] Small I.D., Schallenberg-Rüdinger M., Takenaka M., Mireau H. and Ostersetzer-Biran O. (2020) Plant organellar RNA editing: what 30 years of research has revealed. *Plant J.* 101: 1040–1056. 31630458 10.1111/tpj.14578

[R81] Spåhr H., Chia T., Lingford J.P., Siira S.J., Cohen S.B., Filipovska A., et al. (2018) Modular ssDNA binding and inhibition of telomerase activity by designer PPR proteins. *Nat. Commun.* 9: 2212. 10.1038/s41467-018-04388-1 PMC5992170 29880855

[R82] Sun T., Bentolila S. and Hanson M.R. (2016) The unexpected diversity of plant organelle RNA editosomes. *Trends Plant Sci.* 21: 962–973. 27491516 10.1016/j.tplants.2016.07.005

[R83] Surzycki R., Cournac L., Peltier G. and Rochaix J.-D. (2007) Potential for hydrogen production with inducible chloroplast gene expression in Chlamydomonas. *Proc. Natl. Acad. Sci. U.S.A.* 104: 17548–17553. 17951433 10.1073/pnas.0704205104 PMC2077293

[R84] Takenaka M., Takenaka S., Barthel T., Frink B., Haag S., Verbitskiy D., et al. (2021) DYW domain structures imply an unusual regulation principle in plant organellar RNA editing catalysis. *Nat. Catal.* 4: 510–522. 34712911 10.1038/s41929-021-00633-x PMC7611903

[R85] Takenaka M., Zehrmann A., Brennicke A., Graichen K. and Maas S. (2013) Improved computational target site prediction for pentatricopeptide repeat RNA editing factors. *PLoS One* 8: e65343. 10.1371/journal.pone.0065343 PMC3675099 23762347

[R86] Takenaka M., Zehrmann A., Verbitskiy D., Kugelmann M., Härtel B. and Brennicke A. (2012) Multiple organellar RNA editing factor (MORF) family proteins are required for RNA editing in mitochondria and plastids of plants. *Proc. Natl. Acad. Sci. U.S.A.* 109: 5104–5109. 22411807 10.1073/pnas.1202452109 PMC3324002

[R87] Wang C., Aubé F., Planchard N., Quadrado M., Dargel-Graffin C., Nogué F., et al. (2017) The pentatricopeptide repeat protein MTSF2 stabilizes a nad1 precursor transcript and defines the 3ʹ end of its 5ʹ-half intron. *Nucleic Acids Res.* 45: 6119–6134. 28334831 10.1093/nar/gkx162 PMC5449624

[R88] Wang C., Blondel L., Quadrado M., Dargel-Graffin C. and Mireau H. (2022) Pentatricopeptide repeat protein MITOCHONDRIAL STABILITY FACTOR 3 ensures mitochondrial RNA stability and embryogenesis. *Plant Physiol.* 190: 669–681. 35751603 10.1093/plphys/kiac309 PMC9434245

[R89] Wang C., Lezhneva L., Arnal N., Quadrado M. and Mireau H. (2021) The radish Ogura fertility restorer impedes translation elongation along its cognate CMS-causing mRNA. *Proc. Natl. Acad. Sci. U. S. A.* 118: e2105274118. 10.1073/pnas.2105274118 PMC8536381 34433671

[R90] Weber E., Engler C., Gruetzner R., Werner S., Marillonnet S. and Peccoud J. (2011) A modular cloning system for standardized assembly of multigene constructs. *PLoS One* 6: e16765. 10.1371/journal.pone.0016765 PMC3041749 21364738

[R91] Wu W., Liu S., Ruwe H., Zhang D., Melonek J., Zhu Y., et al. (2016) SOT1, a pentatricopeptide repeat protein with a small MutS-related domain, is required for correct processing of plastid 23S-4.5S rRNA precursors in Arabidopsis thaliana. *Plant J.* 85: 607–621. 26800847 10.1111/tpj.13126

[R92] Yagi Y., Hayashi S., Kobayashi K., Hirayama T., Nakamura T. and Wilusz C.J. (2013) Elucidation of the RNA recognition code for pentatricopeptide repeat proteins involved in organelle RNA editing in plants. *PLoS One* 8: e57286. 10.1371/journal.pone.0057286 PMC3589468 23472078

[R93] Yagi Y., Teramoto T., Kaieda S., Imai T., Sasaki T., Yagi M., et al. (2022) Construction of a versatile, programmable RNA-binding protein using designer PPR proteins and its application for splicing control in mammalian cells. *Cells* 11: 3529. 10.3390/cells11223529 PMC9688318 36428958

[R94] Yang Y., Ritzenhofen K., Otrzonsek J., Xie J., Schallenberg-Rüdinger M. and Knoop V. (2023) Beyond a PPR-RNA recognition code: many aspects matter for the multi-targeting properties of RNA editing factor PPR56. *PLoS Genet.* 19: e1010733. 10.1371/journal.pgen.1010733 PMC10482289 37603555

[R95] Yang F., Vincis Pereira Sanglard C.-P., Lee C.-P., Ströher E., Singh S., Oh G.G.K., et al. (2022) Knockdown of mitochondrial atp1 mRNA by a custom-designed pentatricopeptide repeat protein alters F1-Fo ATP synthase. *Plant Physiol.* (in press). 10.1093/plphys/kiae008 PMC10980415 38206203

[R96] Yan J., Yao Y., Hong S., Yang Y., Shen C., Zhang Q., et al. (2019) Delineation of pentatricopeptide repeat codes for target RNA prediction. *Nucleic Acids Res.* 16: 2089–2011. 10.1093/nar/gkz075 PMC6468296 30753696

[R97] Yan J., Zhang Q., Guan Z., Wang Q., Li L., Ruan F., et al. (2017) MORF9 increases the RNA-binding activity of PLS-type pentatricopeptide repeat protein in plastid RNA editing. *Nat. Plants* 3: 1–8. 10.1038/nplants.2017.37 28394309

[R98] Yin P., Li Q., Yan C., Liu Y., Liu J., Yu F., et al. (2013) Structural basis for the modular recognition of single-stranded RNA by PPR proteins. *Nature* 504: 168–171. 24162847 10.1038/nature12651

[R99] Yoo B.-C., Yadav N.S., Orozco E.M. Jr and Sakai H. (2020) Cas9/gRNA-mediated genome editing of yeast mitochondria and Chlamydomonas chloroplasts. *PeerJ.* 8: e8362. 10.7717/peerj.8362 PMC6951285 31934513

[R100] Yu Q., Barkan A. and Maliga P. (2019) Engineered RNA-binding protein for transgene activation in non-green plastids. *Nat. Plants* 5: 486–490. 31036913 10.1038/s41477-019-0413-0

[R101] Zhelyazkova P., Hammani K., Rojas M., Voelker R., Vargas-Suárez M., Börner T., et al. (2012) Protein-mediated protection as the predominant mechanism for defining processed mRNA termini in land plant chloroplasts. *Nucleic Acids Res.* 40: 3092–3105. 22156165 10.1093/nar/gkr1137 PMC3326301

[R102] Zhou W., Lu Q., Li Q., Wang L., Ding S., Zhang A., et al. (2017) PPR-SMR protein SOT1 has RNA endonuclease activity. *Proc. Natl. Acad. Sci. U.S.A.* 114: E1554–E1563. 28167782 10.1073/pnas.1612460114 PMC5338415

[R103] Zoschke R., Qu Y., Zubo Y.O., Börner T. and Schmitz-Linneweber C. (2013) Mutation of the pentatricopeptide repeat-SMR protein SVR7 impairs accumulation and translation of chloroplast ATP synthase subunits in Arabidopsis thaliana. *J. Plant Res.* 126: 403–414. 23076438 10.1007/s10265-012-0527-1

[R104] Zoschke R., Watkins K.P., Miranda R.G. and Barkan A. (2016) The PPR-SMR protein PPR53 enhances the stability and translation of specific chloroplast RNAs in maize. *Plant J.* 85: 594–606. 26643268 10.1111/tpj.13093 PMC4777676

